# A Deep Sequencing Strategy for Investigation of Virus Variants within African Swine Fever Virus-Infected Pigs

**DOI:** 10.3390/pathogens13020154

**Published:** 2024-02-08

**Authors:** Camille Melissa Johnston, Ann Sofie Olesen, Louise Lohse, Agnete le Maire Madsen, Anette Bøtner, Graham J. Belsham, Thomas Bruun Rasmussen

**Affiliations:** 1 Section for Veterinary Virology, Department of Virus & Microbiological Special Diagnostics, Statens Serum Institute, Artillerivej 5, DK-2300 Copenhagen, Denmark; camj@ssi.dk (C.M.J.); asjo@ssi.dk (A.S.O.); lolo@ssi.dk (L.L.); agnete.madsen@sund.ku.dk (A.l.M.M.); 2 Section for Molecular Ecology and Evolution, Globe Institute, University of Copenhagen, Øster Farimagsgade 5, DK-1353 København, Denmark; 3 Section for Veterinary Clinical Microbiology, Department of Veterinary and Animal Sciences, University of Copenhagen, Stigbøjlen 4, DK-1870 Frederiksberg, Denmark; aneb@sund.ku.dk (A.B.); grbe@sund.ku.dk (G.J.B.)

**Keywords:** African swine fever virus (ASFV), next-generation sequencing (NGS), deep sequencing, nanopore sequencing, minority variant analysis

## Abstract

African swine fever virus (ASFV) is the causative agent of African swine fever, an economically important disease of pigs, often with a high case fatality rate. ASFV has demonstrated low genetic diversity among isolates collected within Eurasia. To explore the influence of viral variants on clinical outcomes and infection dynamics in pigs experimentally infected with ASFV, we have designed a deep sequencing strategy. The variant analysis revealed unique SNPs at <10% frequency in several infected pigs as well as some SNPs that were found in more than one pig. In addition, a deletion of 10,487 bp (resulting in the complete loss of 21 genes) was present at a nearly 100% frequency in the ASFV DNA from one pig at position 6362-16849. This deletion was also found to be present at low levels in the virus inoculum and in two other infected pigs. The current methodology can be used for the currently circulating Eurasian ASFVs and also adapted to other ASFV strains and genotypes. Comprehensive deep sequencing is critical for following ASFV molecular evolution, especially for the identification of modifications that affect virus virulence.

## 1. Introduction

African swine fever virus (ASFV) is the causative agent of African swine fever (ASF), an economically important disease of domestic pigs, often with a high case fatality rate (up to 100%); the virus also infects wild boar and other members of the family *Suidae* [1,2,3,4]. It is the only known DNA arbovirus [2]. The ASFV genome is a large linear double-stranded DNA molecule that ranges in size from 170 to 194 kb depending on the strain and includes almost 200 genes. The genome can be divided into three main regions, with a central conserved region of around 125 kb flanked by two more variable, terminal regions [5].

ASFV exists in multiple genotypes, most of which are confined to Africa [6,7]; however, genotype II was introduced into the republic of Georgia in 2007 [8], and it has since spread through Europe, Asia, and the Caribbean, causing a global pandemic [9,10,11]. The ASFV genome has displayed low diversity among isolates collected over long time periods and from different geographical regions in Europe and Asia [3,9,12,13,14,15,16]. In contrast, greater genetic diversity has been found in ASFVs from East and Southern Africa, likely due to the sylvatic cycle of ASFV within soft ticks and warthogs [3,14,17,18]. While DNA viruses generally have more accurate replication mechanisms than RNA viruses due to the presence of proofreading enzymes, they can still generate genetic diversity through replication errors and recombination events [19,20,21,22,23]. An examination of ASFV samples collected over a period of 70 years revealed evolution rates of approximately 10^−4^ substitutions per nucleotide per year. This rate is closer to that observed in many RNA viruses than is typical for DNA viruses [24]. The DNA polymerase X together with the downstream DNA ligase both exhibit low fidelity, suggesting a potential mutagenic role [25,26]. The quasispecies nature of viral populations has revealed a huge layer of minority genomes, which can modulate the behavior of the viral population or become prominent in response to selective forces or random events, such as bottlenecks [19]. The diversity and quasispecies composition of ASFV has been insufficiently explored, with no known investigations conducted on the impact of quasispecies composition on virulence.

In the present study, we have developed a long-range PCR method, which generates 17 overlapping PCR fragments covering almost the entirety of the ASFV genotype II POL/2015/Podlaskie genome. The PCR products, derived from samples from experimentally infected pigs, were sequenced, and a subsequent variant analysis revealed that the virus present in several pigs exhibited unique SNPs at a 2–10% frequency. All samples were found to have a 10 bp insertion in the intergenic region (IGR) between genes I73R and I329L. The number of occurrences of a 10 bp tandem repeat in this region has been used to further distinguish between ASFV genotype II viruses by dividing them into four different IGR variants: IGR-I with two repeats, IGR-II with three repeats, IGR-III with four repeats, IGR-IV with five repeats [27,28,29]. A large deletion of > 10 kb was present at a nearly 100% frequency in the virus from one infected pig; this was also found to be present at low levels in the virus inoculum and at higher levels in two other infected pigs. This methodology provides the necessary tools for the comprehensive characterization of a virus to explore the influence of viral variants on clinical outcomes and infection dynamics in pigs.

## 2. Materials and Methods

### 2.1. Primers

Primers were designed using the updated ASFV/Georgia/2007 (Accession no. FR682468.2) [30] and ASFV/POL/2015/Podlaskie (Accession no. MH681419.1) [31] reference sequences, covering positions 300-177600 (in Podlaskie) in overlapping regions (Table 1). Two additional primer pairs were designed based on shot-gun sequencing results from DNA isolates from pig 8 to recover information from non-amplifying regions.

### 2.2. Infection Study

Twelve pigs were experimentally infected with the ASFV/POL/2015/Podlaskie, as previously described [32,33]. Briefly, 12 male Landrace x Large White pigs obtained from a conventional Spanish swine herd were challenged intranasally with 4 log_10_ TCID_50_ of the ASFV/POL/2015/Podlaskie (2nd passage) [33]. The pigs were euthanized when reaching the humane end points pre-set in the study: at 6 days post inoculation (dpi) [32]. During the study, EDTA-stabilized blood and serum were obtained from the twelve pigs at 0, 3, 5, and 6 (euthanasia) dpi [32]. At necropsy, spleen material and bone marrow were collected in TriReagent^®^ (Sigma-Aldrich, St. Louis, MO, USA) or phosphate-buffered saline, respectively. The tissue samples were then fast frozen in liquid nitrogen.

**Table 1:** Primer overview.

Name	Fragment	Sequence (5′-3′)
ASFV-300-F ^α^	01_F	AGGTGGTTTGGCCGTATTCT
ASFV-11927-R ^α^	01_R	ATGCGTAGGCCTCCTGAAAG
ASFV-10819-F ^α^	02_F	ATAGGAGCGGCTTGAAGGAC
ASFV-22803-R ^α^	02_R	TGCGGCAACATATGTCCAAAC
ASFV-22129-F ^α^	03_F	CAAAGATGCCGTACCTCCGA
ASFV-33909-R ^α^	03_R	TTTACGGCTTGGGTCAGGAC
ASFV-33237-F ^α^	04_F	GCTCCCTTCAACGCATAGGA
ASFV-44936-R ^α^	04_R	TGCGGGTCTTGGATTAAAGGA
ASFV-43757-F ^α^	05a_F	TGGACCCAAAAAGGGTGGTC
ASFV-56602-R ^α^	05a_R	GCGGCATTGAAAAACACCCT
ASFV-55750-F ^α^	05b_F	TGAGCTGTTCCCAGGATTCG
ASFV-68434-R ^α^	05b_R	AGCGCGCGTATTTATCAACG
ASFV-67660-F ^β^	06_F	GGACTGCGACACGATCACAGAGTC
ASFV-78076-R ^β^	06_R	GTCCTACGACACCATGCGAACCAAG
ASFV-77808-F ^β^	07_F	CTATATTGGCAAACTGTTTCACGTC
ASFV-87981-R ^θ^	07_R	CAATCACAACGGTTCTCCTGTTAAG
ASFV-87000-F ^α^	08_F	GCATAATCAATGGCAATCCCCC
ASFV-97970-R ^α^	08_R	TGGCCTTAATCATTACAGCGGT
ASFV-97345-F ^β^	09_F	GTTACGTAGATCACTGAGTTGCAATC
ASFV-107683-R ^β^	09_R	GGCGCCCTCCTATACGATGAG
ASFV-107531-F ^β^	10_F	GTGTCCTCCATCGGATATACTATAC
ASFV-117620-R ^θ^	10_R	AGTGTGCTGACCTATATCACGGAAC
ASFV-117410-F ^β^	11_F	CATTTCTGAACTGCGAGAGTTCTAG
ASFV-127433-R ^β^	11_R	TCGCTGTGCGTAATTTATCCCAATC
ASFV-126429-F ^α^	12_F	AACACCTAACCTCGTCGTGC
ASFV-137952-R ^α^	12_R	ACAGGTAAGGTCCGACTCGT
ASFV-137097-F ^β^	14_F	GAGAACAGGTCTTAGAATTACTTCATG
ASFV-147178-R ^β^	14_R	ACGCATCCGAAGGTGTTACAAGGAC
ASFV-146744-F ^θ^	15_F	CTCTGAATGCGCAGAGCATCTTAC
ASFV-156426-R ^β^	15_R	GAACATGGGAATACGTGTGTCCAG
ASFV-155654-F ^α^	17_F	AGGAACTGGACATGCAAGCAG
ASFV-166805-R ^α^	17_R	ATGAGCTCGCCCACATAACC
ASFV-166089-F ^α^	18_F	TATTGCCCGAGCCTCTGTATTC
ASFV-177600-R ^α^	18_R	GGGGGAATCAACTCTCGCTTAA
ASFV-6188-F ^α^	del-PCR_F	GCTTCTAACTCTCTGTACAACA
ASFV-17145-R ^α^	del-PCR_R	CGGCATATCATAAGTAGGTTGGT
ASFV-6708-F ^α^	noDel-PCR_F	AAGTGGCTGCTCGTCAACAA
ASFV-7668-R ^α^	noDel-PCR_R	AGCCGTAGCAATGTTGGTGA

^α^ Designed in this study; ^β^ Designed by Portugal et al. [34]; ^θ^ Modified from Portugal et al. [34]

### 2.3. Preparation of Long PCR Products from Viral DNA

DNA was extracted from serum, EDTA blood, spleen, and bone marrow samples of individual pigs from the infection study (collected on the day of euthanasia) as well as from the virus sample used as the inoculum (1st and 2nd passage) (Table 2). This was done using a MagNA Pure 96 system (Roche, Basel, Switzerland) with the DNA/Viral NA 2.0 kit and the Viral NA Plasma external lysis S.V. 3.1. protocol. In short, spleen and bone marrow homogenate suspensions (25% *w*/*v*) were prepared in Minimum Essential Medium (Gibco, Thermo Fisher Scientific, Waltham, MA, USA). The samples were homogenized using two 3 mm stainless steel beads (Dejay Distribution Ltd., Launceston, UK) in a TissueLyser II (QIAGEN, Hilden, Germany). The homogenates were centrifuged and supernatants collected for DNA purification. The extracted samples were analyzed for the presence of ASFV DNA with qPCR using the CFX Opus Real-Time PCR System (Bio-Rad, Hercules, CA, USA), essentially as described by Tignon et al. [35]. Viral load of ASFV is given as Ct values, and a positive result is defined as giving a Ct value below 42.

The extracted DNA preparations from ASFV DNA positive samples were used to generate overlapping long PCR products, ranging in size from approx. 9600 bp to 12,800 bp, employing a modified version of the long PCR method described previously [36]. Briefly, the samples, except for those from serum, were diluted 1:10 in UltraPure™ DNase/RNase-Free Distilled Water (Invitrogen, Thermo Fisher Scientific, Waltham, MA, USA). The products were amplified by long PCR using AccuPrime high-fidelity DNA polymerase (Thermo Scientific, Thermo Fisher Scientific, Waltham, MA, USA) in a final volume of 50 μL using 94 °C for 30 s followed by 35 cycles of 94 °C for 15 s, 55 °C for 30 s, and 68 °C for 12 min and a final extension of 68 °C for 12 min. As a positive control, we used extracted DNA from the spleen of an ASFV-infected pig, derived from a previous study [37], and as negative control, we used UltraPure™ DNase/RNase-Free Distilled Water. The PCR products were analyzed using the Genomic DNA ScreenTape on a 4200 TapeStation (Agilent Technologies, Santa Clara, CA, USA) together with GeneRuler 1 kb Plus DNA Ladder (Thermo Scientific), and their concentrations were estimated using either the Qubit™ 1× dsDNA broad-range kit (Invitrogen) with the Qubit™ Fluorometer (Invitrogen) or Quant-iT™ 1× dsDNA broad-range kit (Invitrogen) on a FLUOstar® Omega (BMG LABTECH, Mornington, VIC, Australia) instrument.

### 2.4. Next Generation Sequencing (NGS)

PCR products were pooled for each sample and sequenced by NGS (incl. appropriate controls) using MiSeq (Illumina, San Diego, CA, USA) with a modified Nextera XT DNA library protocol with the MiSeq reagent kit v2 (300 cycles), resulting in 2× 150 bp paired-end reads. The PCR products were also sequenced on the Nanopore (Oxford Nanopore Technologies, Oxford, UK) using a standard ligation sequencing of amplicons with native barcoding protocol for the SQK-LSK109 and native barcoding expansion kits on a R9.4.1 flow-cell. MiSeq reads were trimmed using AdapterRemoval [38] by at least 30 bp at both the 5′ and 3′ ends to ensure primer removal as well as for quality (q30) using a sliding window. Some samples were sequenced directly (no PCR amplification) using the MiSeq as described above. Nanopore reads were trimmed using chopper [39] by 30 bp at both the 5′ and 3′ ends as well.

### 2.5. PCR Deletion Screening and Sanger Sequencing

Long PCRs were performed as described above on ASFV DNA positive samples with primers spanning the deletion. The PCR products were analyzed, as above, using the Genomic DNA ScreenTape or D5000 ScreenTape, and their concentrations were estimated using a fluorometer as above. PCR products were purified using the GeneJET PCR Purification Kit (Thermo Scientific) according to the manufacturer’s instructions and were then sequenced using the Sanger system with a combination of BigDye Terminator v. 1.1. Cycle Sequencing Kit (Applied BioSystems, Waltham, MA, USA) with 10 µM primers, purification using SigmaSpin Post-Reaction columns (Sigma-Aldrich, St. Louis, MO, USA), and an ABI3500 Genetic Analyzer (Applied BioSystems). Sequences were analyzed using Geneious (Biomatters INC., Boston, MA, USA).

### 2.6. Variant and Indel Calling

MiSeq reads were mapped to the ASFV/POL/2015/Podlaskie reference sequence using BWA-MEM [40], and aligned reads were filtered for mapping quality (mapq) 60; secondary and supplementary reads were removed using Samtools [41]. Variant calling and annotation was performed using a combination of Lo-Freq [42] and SnpEFF [43] as described previously [44]. Briefly, quality scores were recalibrated by Lo-Freq, and variants were filtered for a minimum coverage of 100, frequency above 2%, and strand-bias Phred Score below 12. For all variants that successfully passed filtering in a particular sample, their presence in other samples was examined, even at below specified thresholds, and annotations were applied accordingly. A custom script was made to scan variants for homopolymer runs; a homopolymer was defined as 4 or more consecutive repeated nucleotides.

Insertions and deletions (indels) were also called for the Nanopore reads obtained from each sample by mapping the reads to the ASFV/POL/2015/Podlaskie reference sequence using a combination of minimap2 [45] and Samtools. The alignment was first filtered to select reads that spanned the entirety of each PCR region. Up to 1000 reads were extracted for each PCR product from the alignment. The Compact Idiosyncratic Gapped Alignment Report (CIGAR) strings, which consist of matches (M), insertions (I), and deletions (D) [46], of the alignment files were parsed with a custom script to detect indels above 5 bp. Strand bias was calculated using Fisher’s exact test, and *p*-values were transformed to Phred scale. Indels were filtered for a minimum of 5 reads present on both forward and reverse strands and strand-bias Phred Score below 12 as well as a frequency ≥2%. Variant annotation was performed using SnpEFF, and indels were scanned for homopolymer runs as described above.

## 3. Results

### 3.1. Infection Study

**Figure 1:**
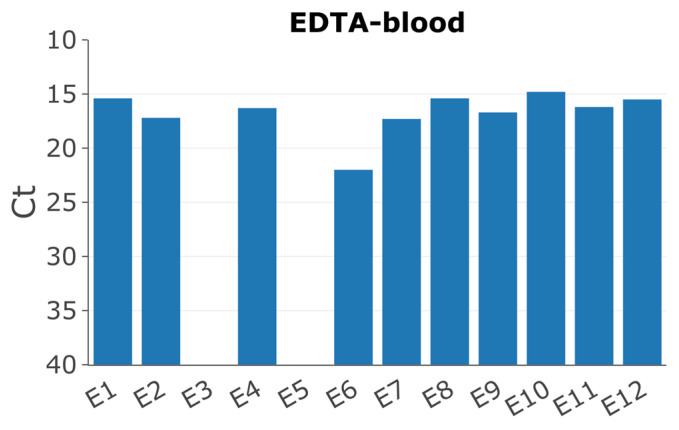
Detection of ASFV DNA by qPCR [35] in EDTA blood of the different pigs collected on day of euthanasia (PID 6).

Twelve pigs had been experimentally inoculated with the ASFV/POL/2015/Podlaskie isolate as previously described [32]. Pigs 3 and 5 showed no clinical signs of disease; furthermore, the qPCRs to detect ASFV DNA in EDTA blood and serum were negative (Figure 1), and hence, samples from these pigs were excluded from this study. Pig 6 displayed delayed onset of symptoms, consistent with the higher Ct value of 22 for the EDTA blood sample compared to the other pigs, which all displayed high viral loads with Ct values between approx. 15-17 (Figure 1).

### 3.2. Overlapping Long PCRs

**Figure 2:**
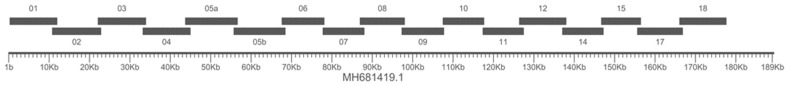
PCR schematic of the overlapping long PCRs.

We designed 17 overlapping primer sets covering 93.6% of the ASFV/POL/2015/Podlaskie reference genome (Figure 2). We initially used serum samples for long PCR amplification; however, the yield of products was low compared to our positive control. Therefore, we performed a dilution series on the four sample types (EDTA blood, bone marrow, spleen, and serum) to determine the optimum DNA input for the long PCRs (Table 3). Serum samples generated less product overall than the other sample types, whereas DNA isolated from EDTA blood was able to generate the expected products at different yields corresponding to the dilution. Bone marrow and spleen samples both failed to generate products when used undiluted. Hence, the EDTA blood samples collected at euthanasia and the virus inoculum were chosen for the initial analyses. Long PCRs were performed on the inoculum and 10x diluted EDTA blood samples, and the products were sequenced by MiSeq, except for those from pig 6, which performed poorly in the PCR amplification, likely due to its lower concentration (higher Ct value), and it was therefore excluded from further study. In contrast to the other samples, the samples from pig 8 consistently failed to yield PCR products for fragments 01 and 02 in all tissue types, although the other fragments were successfully generated from these samples.

### 3.3. Identification of a Large Deletion Event in the ASFV Genomes within Pig 8

**Figure 3:**
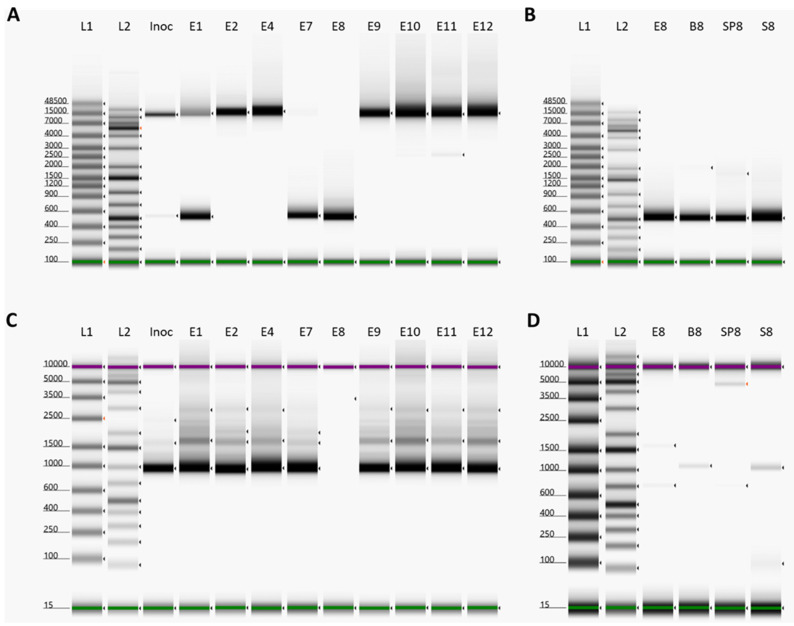
Deletion screening on samples collected on day of euthanasia. (**A**) PCR with primers covering nt 6188-17145. L1: Genomic Ladder, L2: GeneRuler 1kb plus, Inoc: Inoculum, E1: Pig 1 EDTA-blood, E2: Pig 2 EDTA-blood, E4: Pig 4 EDTA-blood, E7: Pig 7 EDTA-blood, E8: Pig 8 EDTA-blood, E9: Pig 9 EDTA-blood, E10: Pig 10 EDTA-blood, E11: Pig 11 EDTA-blood, E12: Pig 12 EDTA-blood. (**B**) PCR with primers covering nt 6188-17145. L1: Genomic Ladder, L2: GeneRuler 1kb plus, E8: Pig 8 EDTA-blood, B8: Pig 8 bone-marrow, SP8: Pig 8 spleen, S8: Pig 8 serum. (**C**) PCR with primers covering nt 6708-7668. L1: D5000 Ladder, L2: GeneRuler 1kb plus, Inoc: Inoculum, E1: Pig 1 EDTA-blood, E2: Pig 2 EDTA-blood, E4: Pig 4 EDTA-blood, E7: Pig 7 EDTA-blood, E8: Pig 8 EDTA-blood, E9: Pig 9 EDTA-blood, E10: Pig 10 EDTA-blood, E11: Pig 11 EDTA-blood, E12: Pig 12 EDTA-blood. (**D**) PCR with primers covering nt 6708-7668. L1: D5000 Ladder, L2: GeneRuler 1kb plus, E8: Pig 8 EDTA-blood, B8: Pig 8 bone-marrow, SP8: Pig 8 spleen, S8: Pig 8 serum. Green and purple bands indicate lower and upper molecular weight markers, respectively, whereas arrows indicate bands detected by the TapeStation Analysis software.

Due to the failure of DNA isolated samples from pig 8 to yield PCR products for fragments 01 and 02, EDTA blood, bone marrow, and spleen samples of pig 8 were sequenced directly by Illumina MiSeq. No reads were present that mapped to pos. 6364-16878 of the ASFV Podlaskie reference sequence; therefore, we designed primers that spanned across this region at pos. 6188-17145 (del-PCR). The use of these primers generated a PCR product of ~500 bp in all of the pig 8 samples (Figure 3B). The EDTA blood samples from the other pigs and from the inoculum were screened for the presence of this deletion. Samples from pigs 1 and 7 also displayed prominent products of ~500 bp, consistent with the presence of the deletion, while the inoculum displayed a very weak product of this size (Figure 3A). All samples except those from pig 7 and pig 8 also yielded products at ~11 kb, consistent with the expected full-length PCR products (without the deletion). The ca. 500 bp PCR product from pig 8 was sequenced using the Nanopore and Sanger systems, which each revealed that the deletion occurred between positions 6362 and 16849, a total of 10,487 bp. This deletion results in the loss of 21 complete genes; the majority are members of the MGF 110 (2L-14L) family, with a 3′ truncation of the MGF 110-1L gene and ASFV G ACD 00090, 00120, 00160, 00190, 00240, and 00270 as well as MGF 360-4L. To confirm the deletion of nucleotides 6362-16849, we designed primers located within this region at pos. 6708-7668 (noDel-PCR) and screened all samples as above. All samples except those from pig 8 yielded the expected product at ~1000 bp (Figure 3C), consistent with the ability to produce full-length PCR fragments 01 and 02. The screening of the other samples from pig 8 revealed a very weak ~1000 bp product in the bone marrow and serum samples (Figure 3D).

In order to elucidate when this deletion variant arose in the inoculum, the first passage sample of the ASFV stock was also screened using these PCRs. They yielded only a full-length PCR product for the del-PCR and the expected ~1000 bp product for the noDel-PCR (Figure S1), indicating that the deletion event took place during the production of the second passage virus stock in porcine pulmonary alveolar macrophages (PPAM), which was used as the inoculum in the infection study.

### 3.4. Variant Analysis of EDTA Blood Samples

All samples were screened for the presence of SNPs and indels in the MiSeq reads as well as indels above 5 bp in the full-length Nanopore reads derived from the various PCR products in order to detect larger deletions.

The identified 10,487 bp deletion in the ASFV DNA from pig 8, which spans the region 6362-16849, results in the failure of the PCRs that generate fragments 01 and 02. This is due to the locations of their reverse and forward primers, respectively, within the deleted region. Consequently, only full-length versions of this region of the genome are successfully amplified. This means that the large deletion in the virus from pig 8 (and pigs 1 and 7) does not appear in this indel analysis. However, for pigs 1 and 7, the undeleted form of the virus was also present; therefore, it was possible to assess changes within these regions of the genome in these samples, but it was not possible for pig 8.

**Figure 4:**
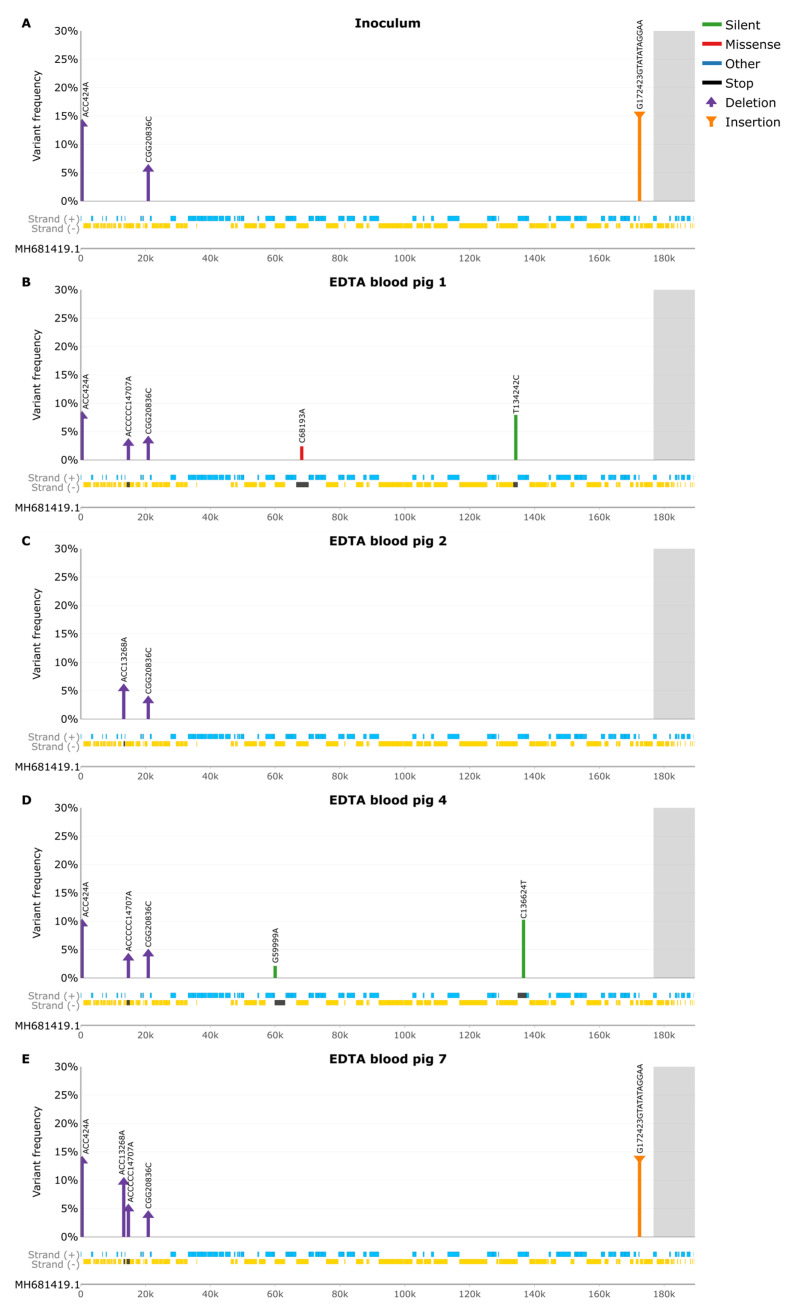

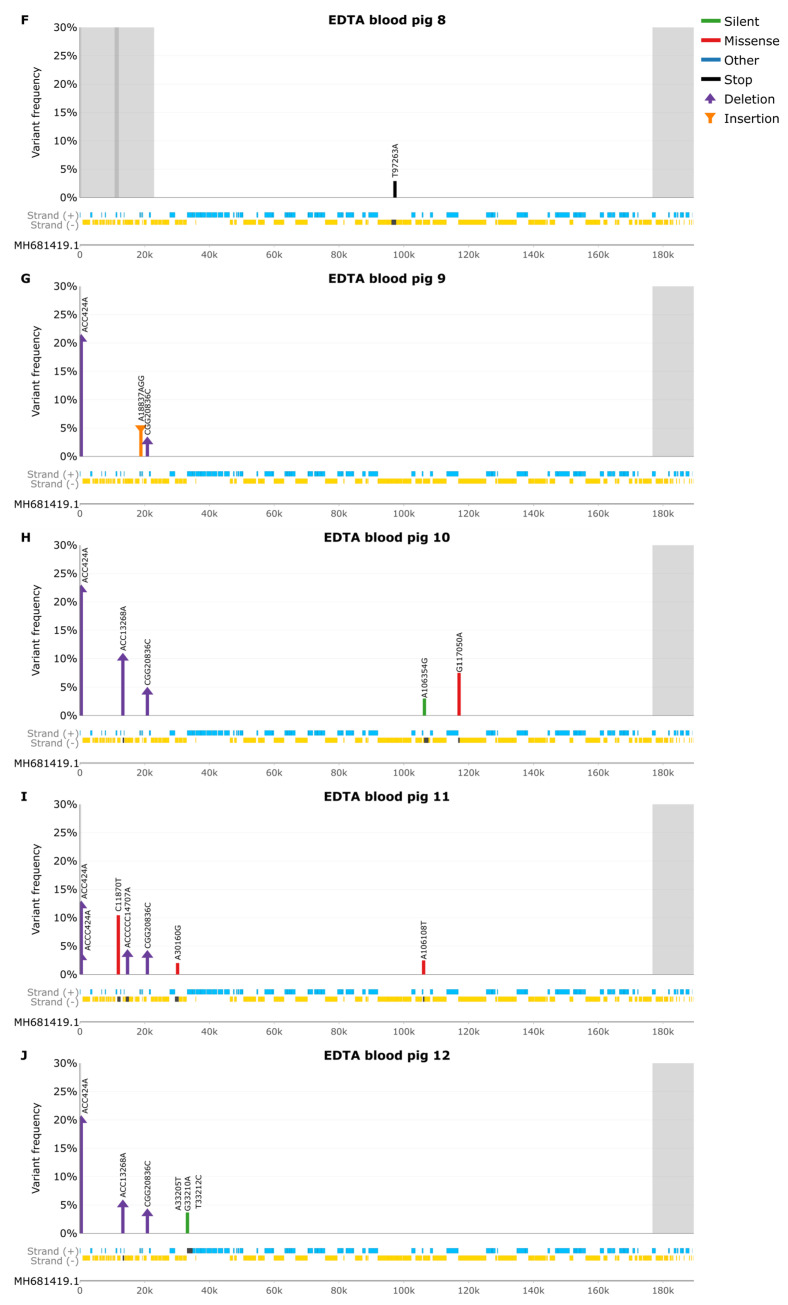
Variant frequency (Lo-Freq) plots of silent (green), missense (red), other (blue), insertion (purple), and deletion (orange) mutations. Grey background indicates areas with no PCR coverage. Genes on the forward strand are indicated in blue, and genes on the reverse strand are indicated in gold, with affected genes in dark grey. (**A**) SNP frequencies in Inoculum. (**B**) SNP frequencies in EDTA blood pig 1. (**C**) SNP frequencies in EDTA blood pig 2. (**D**) SNP frequencies in EDTA blood pig 4. (**E**) SNP frequencies in EDTA blood pig 7. (**F**) SNP frequencies in EDTA blood pig 8. (**G**) SNP frequencies in EDTA blood pig 9. (**H**) SNP frequencies in EDTA blood pig 10. (**I**) SNP frequencies in EDTA blood pig 11. (**J**) SNP frequencies in EDTA blood pig 12.

In the virus inoculum, a total of three indels were present, one a 2 bp deletion at a 13.6% frequency located at nt 424 in a non-coding homopolymeric region (Figure 4A and Table S1). This deletion was shared among all EDTA blood samples except for pig 2 and pig 8, ranging in frequencies between 7.7% and 22.2% (Table S2). The second indel was also a 2 bp deletion located at nt 20836 in a non-coding homopolymeric region, with a frequency of 5.6%. This deletion was also present in all other EDTA blood samples except for pig 8 at a 2.6–4.3% frequency. The third indel in the inoculum was a 10 bp insertion, a tandem repeat ‘TATATAGGAA’, at 172423, which was located in a non-coding region between the I73R and I329L genes with a frequency of 15.3%, and it was found also in the EDTA blood obtained from pig 7 at 13.8% frequency. This insertion was also detected in all the other EDTA blood samples from the remaining ASFV-infected pigs at 12.2–23.7% frequencies, although these did not pass the strand-bias requirements. This insertion was also found in the Nanopore indel analysis, though at higher frequencies, ranging from 32.5% to 44.3% in all samples (some failed due to strand bias) (Table S3). Due to this discrepancy, we counted the number of tandem repeats in the trimmed reads (pre-alignment), which revealed that 96.3–100% of the reads in both the MiSeq and Nanopore reads corresponded to the IGR-II variant. The disparity between the raw reads and alignment is due to reference bias (see Discussion). The published ASFV/POL/2015/Podlaskie reference strain sequence [31] does not contain this tandem repeat; however, the results from this current study demonstrate that the ASFV/POL/2015/Podlaskie strain is actually an IGR-II variant. This disparity is discussed below.

The virus in EDTA blood obtained from pig 1 (Figure 4B) had two unique SNPs, a missense and silent mutation, C68193A and T134242C, located in the EP1242L (A693S) and NP419L genes, respectively, at a 2.4% and 7.9% frequency respectively (Table S1). It also contained one unique 5 bp deletion at nt 14707 with a frequency of 3%, causing a frameshift within the MGF 110-13Lb gene (Table S2). In the sample from pig 2 (Figure 4C), there was one 2 bp deletion located in a homopolymeric region, causing a frameshift in the MGF 110-10L (nt 13268) gene, at a 5.4% frequency (Table S2). This deletion was also found in the EDTA blood of pigs 7, 10, and 12 at 5-10.1% frequencies. It was also present, although it did not pass the strand-bias requirements, in all the remaining samples at 6.3-13.9% frequency except for pig 8.

The virus in the EDTA blood obtained from pig 4 (Figure 4D) had two unique silent SNPs, G59999A and C136624T located in the F1055L and NP868R genes, at 2.1% and 10.3% frequency, respectively (Table S1). In contrast to all other samples, the virus in the sample from pig 7 (Figure 4E) did not have any unique variants called by the Lo-Freq variant analysis (Table S2); however, the Nanopore indel analysis revealed a 5 bp insertion at nt 14707 causing a frameshift in the MGF 110-13Lb ORF with a frequency of 16.6%, which was also present in pig 9 at 12.3% (Table S3).

The ASFV in blood obtained from pig 8 (Figure 4F) had a total of one unique nonsense SNP, T97263A (K99*), causing an early stop codon in the B438L ORF, at a 2.9% frequency (Table S1). In the EDTA blood from pig 9 (Figure 4G), the ASFV DNA had one unique 2 bp insertion in a non-coding homopolymeric region, with a frequency of 5% (Table S2). The virus in the blood from pig 10 (Figure 4H) had two SNPs, one silent and one missense, A106354G and G117050A (P46S), located in the B407L and CP123L genes with frequencies of 3% and 7.5%, respectively (Table S1). The G117050A was also present in pig 11, though it did not pass the filtering requirements.

The virus population in pig 11 (Figure 4I) showed three missense SNPs, C11870T (D213N), A30160G (W97R), and A106108T (N63K), affecting the MGF 110-9L, MGF 306-12L, and B117L genes at 10.4%, 2%, and 2.5% frequencies, respectively (Table S1). The A30160G SNP was also present in pigs 1, 4, 7, 8, 9, 10, and 12, though these did not pass filtering requirements. Additionally, there was also a 3 bp deletion present at nt 424 in a non-coding homopolymeric region with a frequency of 3% (Table S2).

The ASFV genomes within pig 12 (Figure 4J), displayed three unique SNPs, two silent and one missense, located at A33205T, G33210A (R26Q), and T33212C, all in the MGF 505-2R gene with frequencies ranging from 3.4% to 3.7% (Table S1). These three SNPs were also present in pig 10, though they did not pass filtering requirements.

### 3.5. Samples from Pig 2

**Figure 5:**
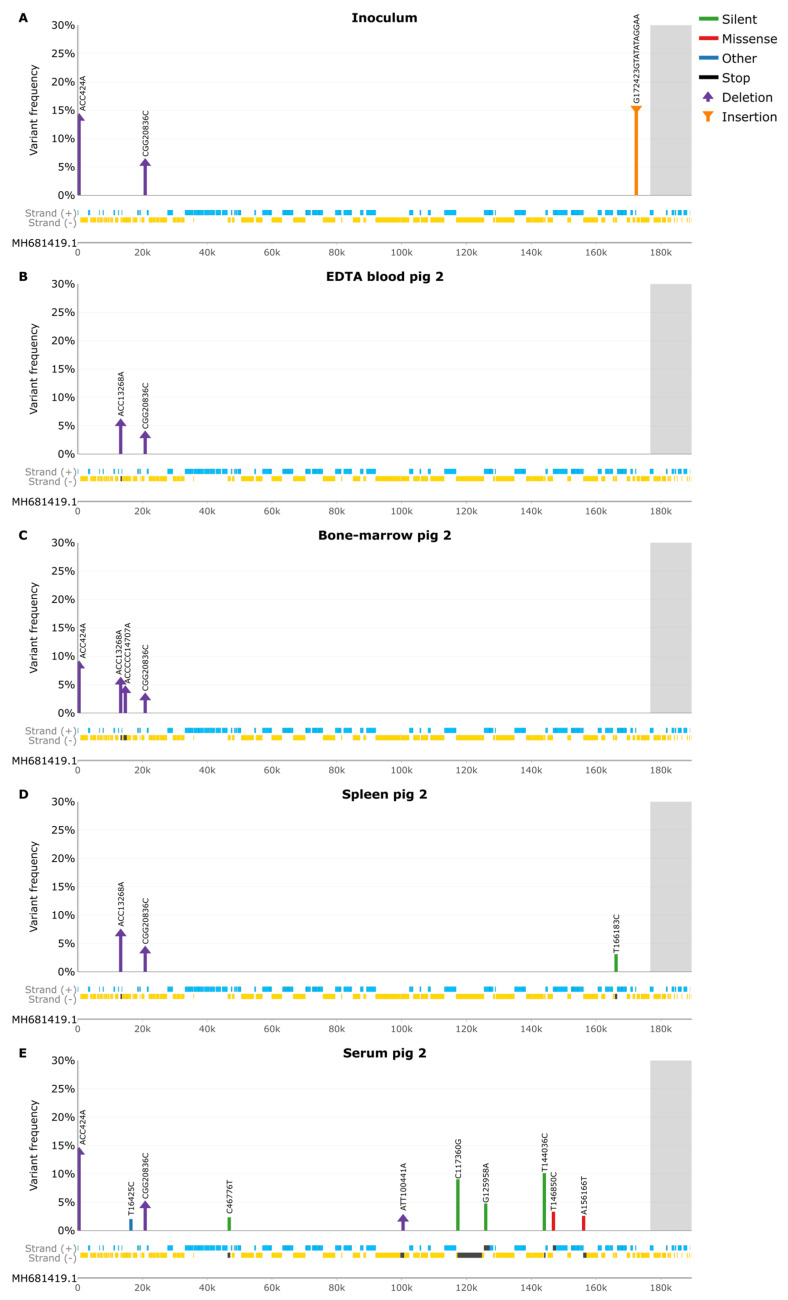
Variant frequency (Lo-Freq) plots of silent (green), missense (red), other (blue), insertion (purple), and deletion (orange) mutations. Grey background indicates areas with no PCR coverage. Genes on the forward strand are indicated in blue, and genes on the reverse strand are indicated in gold, with affected genes in dark grey. (**A**) SNP frequencies in Inoculum. (**B**) SNP frequencies in EDTA blood pig 2. (**C**) SNP frequencies in bone marrow pig 2. (**D**) SNP frequencies in spleen pig 2. (**E**) SNP frequencies in serum pig 2.

To explore virus tropism across various tissues from an individual pig, pig 2 was arbitrarily chosen for further investigation (Figure 5). As mentioned above, the virus inoculum had three indels (Figure 5A). The deletion at nt 424 was also present in the bone marrow (Figure 5C) and serum (Figure 5E) samples of pig 2 at 8.3% and 13.9% frequency (Table S5), whereas the deletion at nt 20836 was present in all pig 2 tissues, ranging in frequencies from 2.7% to 4.4%. The 10 bp insertion was also present in all tissues, though this failed due to strand bias in the Lo-Freq, called variants, but it was found at high frequencies in the Nanopore indel analysis (Table S6). As mentioned above, the EDTA blood sample from pig 2 (Figure 5B) contained a 2 bp deletion at nt 13268, which was also found in all other pig 2 tissues (some did not pass filtering) at similar frequencies (Table S5). The bone marrow sample of pig 2 had a unique 5 bp deletion at nt 14707 located in a homopolymer region, causing a frameshift in the MGF 110-13Lb ORF at a 4% frequency.

The spleen sample (Figure 5D) obtained from pig 2, displayed one unique silent SNP, T166183C located in E199L at a 3% frequency (Table S4), whereas the serum sample showed seven SNPs. The four silent SNPs, C46776T, C117360G, G125958A, and T144036C in the A224L, CP2475L, CP530R, and D177L genes, ranged in frequencies from 2.3% to 10.1%. The C46776T SNP was also present in the inoculum and EDTA blood of pig 2 but did not pass filtering conditions. The two missense SNPs, T146850C (S47P) and A156166T (F276I), affecting the S237R and R298L genes had frequencies of 3.3% and 2.6%, respectively. The final SNP, T16425C, located in a non-coding region with a frequency of 2%, was also present in the other pig 2 tissue samples, but these did not pass filtering requirements. Additionally, the serum sample displayed a 2 bp deletion at nt 100441, located in a homopolymeric region, causing a frameshift within B354L, with a frequency of 2% (Table S5). This deletion was also found in the other pig 2 tissues, but these did not pass filtering conditions.

## 4. Discussion

Using a deep-sequencing strategy on ASFV DNA samples derived from experimentally infected pigs, we have identified a variety of changes within the viral genome that have occurred during replication in the pigs. The largest change was a deletion of over 10 kb, which initially arose in cell culture in generating the inoculum but became dominant in pig 8 and was present in pigs 1 and 7. This large deletion (pos. 6362 to 16849) in the ASFV DNA in pig 8 completely removes 21 genes and truncates another gene. The deletion did not prevent the production of severe disease, as the pig was still clinically ill [32], and high levels of β-actin DNA were still circulating in its serum [47]. However, the effect of such deletions on the transmission to a new host or its ability to grow in arthropod vectors is unknown, but there must have been a significant advantage for the deletion to have taken over nearly the entire virus population within this pig. Deletions in this part of the genome have also happened in the field, e.g., the majority of the lost genes in pig 8 are shared with the Estonia2014 strain (see [48]), except for ASFV G ACD 00270, MGF 110-13Lb, and MGF 360-4L, which are still present; however, MGF 110-13La is truncated at the 3’-end. Estonia2014 is characterized as having reduced virulence; it additionally lacks KP177R, L83L, L60L, and MGF 360 (1L-3L) compared to the deletion within the ASFV DNA in pig 8. The MGF360-1L and MGF110-1L genes do not seem to be involved in virulence in swine [49,50], and similarly, KP177R, which encodes the structural protein p22, does not seem to be involved in determining virulence in swine [51]. L83L is a nonessential protein and does not affect pathogenicity in vitro or in vivo; however, it seems to play a role in the inhibition of the type I interferon production [52,53]. These results suggest that the L60L gene could serve as a virulence determinant [54].

Limited information is available regarding the majority of the genes deleted in the ASFVs in samples from pig 8. The MGF110-5L-6L gene appears not to play a role in virus replication or virulence in swine [55], whereas MGF110-9L does seem to play such a role [56]. The MGF110-7L is implicated in subverting the host protein synthesis machinery, inducing phosphorylation of eIF2α through protein kinase R (PKR) and PKR-like endoplasmic reticulum (ER) kinase (PERK). This process was found to be essential for host translation repression and stress granule (SG) formation [57]. Certain members of the MGF360 family have been identified as essential for replication in pigs, ticks, and macrophages [58,59,60,61,62,63,64].

The complete switch in the virus population observed in pig 8 from the deletion variant being a small component of the population in the inoculum, as evident by the generation of a mostly full-length product, to being the dominant virus was very apparent (see Figure 3A,C). This indicates that a minority variant can become dominant in just a single round of infection within pigs. Thus, potentially, a minor variant of the initial virus population can change into the major source of infection from a particular infected pig. Hence, the properties of the minority variant virus can become the properties of the circulating virus, and the nature of the disease will reflect the properties of the new variant. Transmission of the virus from one host animal to another will often involve some sort of bottleneck event, and a minority variant can then become a major component within some of the newly infected animals even without a major selection advantage (as observed here and previously with classical swine fever virus (CSFV) [44]).

Mostly minor SNP variations were observed between the different sample types obtained from pig 2. The serum sample contained many more SNPs than the other samples; however, this is to be expected, as the virus in serum sample likely represents a compilation of all the viruses present in the various tissues from pig 2.

Recently, 24 genetic groups have been identified within the genotype II ASFVs, which have been circulating in Europe, using a multi-gene approach to subtyping [28]. These variants differ by a variety of changes, including SNPs and deletions in the central variable region (CVR variants), tandem repeat sequences (TRS) in the intergenic region (IGR) between genes I73R/I329L (IGR variants), a 14 nt insertion in the O174L gene (O174 variants), a SNP in the K145R gene (K145R variants), SNPs in the IGR between the I329L and I215L genes, and the I215L gene itself (ECO2 variants). In addition, there are differences in the TRS in the IGR between the MGF505-9R and the MGF505-10R genes (MGF variants). Using this nomenclature system, the published sequence for ASFV/POL/2015/Podlaskie would be classified as an CVR-I, IGR-I, O174L-I, K145R-I, MGF-I, and ECO2-I variant. However, the IGR-II variant has been found in the majority of genotype II strains circulating in Europe [28]. In the studies presented here, it is apparent that the ASFV/POL/2015/Podlaskie in the inoculum is also an IGR-II variant and that the published ASFV/POL/2015/Podlaskie reference strain should be re-classified as an IGR-II variant, which places the ASFV/POL/2015/Podlaskie isolate in genetic group 3 [28].

The complete genome sequence of ASFV/POL/2015/Podlaskie was originally generated using a reference-based alignment to the ASFV/Georgia/2007 reference strain [31]. The results of our study emphasize the importance of the awareness of reference bias, where reads harboring non-reference genomic variations, can be inaccurately aligned or overlooked by the aligner [65,66].

Nanopore sequencers are known to struggle to accurately sequence homopolymers due to the lack of a signal change [67]. Previous studies have found that about 47% of errors were linked to homopolymers [68]. The new Nanopore R10.4 flow cells are more accurate in calling homopolymers in the 4–9 bp range than the R9.4.1 flow cells [69], which we have used in this current study. The majority of the indels reported in this study are associated with homopolymers, and it is difficult to determine their veracity by other means, as most sequencing technologies have difficulties with homopolymers [70,71,72,73].

Comprehensive deep sequencing plays a pivotal role in following ASFV molecular evolution and pinpointing modifications that may influence virulence. Given the substantial size of the ASFV genome and usually an unfavorable virus-to-host ratio, achieving the required ASFV DNA read numbers for high-quality whole-genome sequencing consistently demands a significant amount of sequencing power when using direct sequencing. This current methodology facilitates high-throughput sequencing with a large number of samples, as done in this study; variant calling; and indel detection, with the flexibility to be tailored for other ASFV strains and genotypes by modifying primers in regions with low compatibility. This approach is suitable for the current ASFVs circulating in Eurasia and provides a foundation for understanding ASFV evolution.

## Figures and Tables

**Table 2:** Sample overview.

Sample ID ^α^	Sample Name	Matrix	Ct ^β^
Spleen, 1/50 2. passage LPPAM 02-07-2019	Inoc	Cell culture supernatant	25.2
CReSA_2020_pig_1_eut.	S1	Serum	20.2
CReSA_2020_pig_1_eut.	E1	EDTA-blood	15.4
CReSA_2020_pig_1_eut.	B1	Bone-marrow ^θ^	19.0
CReSA_2020_pig_1_eut.	SP1	Spleen ^θ^	15.6
CReSA_2020_pig_2_eut.	S2	Serum	19.3
CReSA_2020_pig_2_eut.	E2	EDTA-blood	17.2
CReSA_2020_pig_2_eut.	B2	Bone-marrow ^θ^	16.8
CReSA_2020_pig_2_eut.	SP2	Spleen ^θ^	15.7
CReSA_2020_pig_3_eut.	S3	Serum	No Ct
CReSA_2020_pig_3_eut.	E3	EDTA-blood	No Ct
CReSA_2020_pig_3_eut.	B3	Bone-marrow ^θ^	38.3
CReSA_2020_pig_3_eut.	SP3	Spleen ^θ^	39.5
CReSA_2020_pig_4_eut.	S4	Serum	19.2
CReSA_2020_pig_4_eut.	E4	EDTA-blood	16.3
CReSA_2020_pig_4_eut.	B4	Bone-marrow ^θ^	19.1
CReSA_2020_pig_4_eut.	SP4	Spleen ^θ^	16.2
CReSA_2020_pig_5_eut.	S5	Serum	No Ct
CReSA_2020_pig_5_eut.	E5	EDTA-blood	No Ct
CReSA_2020_pig_5_eut.	B5	Bone-marrow ^θ^	No Ct
CReSA_2020_pig_5_eut.	SP5	Spleen ^θ^	39.8
CReSA_2020_pig_6_eut.	S6	Serum	29.0
CReSA_2020_pig_6_eut.	E6	EDTA-blood	22.0
CReSA_2020_pig_6_eut.	B6	Bone-marrow ^θ^	22.3
CReSA_2020_pig_6_eut.	SP6	Spleen ^θ^	16.5
CReSA_2020_pig_7_eut.	S7	Serum	20.5
CReSA_2020_pig_7_eut.	E7	EDTA-blood	17.3
CReSA_2020_pig_7_eut.	B7	Bone-marrow ^θ^	19.3
CReSA_2020_pig_7_eut.	SP7	Spleen ^θ^	16.5
CReSA_2020_pig_8_eut.	S8	Serum	19.4
CReSA_2020_pig_8_eut.	E8	EDTA-blood	15.4
CReSA_2020_pig_8_eut.	B8	Bone-marrow ^θ^	19.1
CReSA_2020_pig_8_eut.	SP8	Spleen ^θ^	15.8
CReSA_2020_pig_9_eut.	S9	Serum	19.7
CReSA_2020_pig_9_eut.	E9	EDTA-blood	16.7
CReSA_2020_pig_9_eut.	B9	Bone-marrow ^θ^	19.2
CReSA_2020_pig_9_eut.	SP9	Spleen ^θ^	17.3
CReSA_2020_pig_10_eut.	S10	Serum	19.3
CReSA_2020_pig_10_eut.	E10	EDTA-blood	14.8
CReSA_2020_pig_10_eut.	B10	Bone-marrow ^θ^	18.2
CReSA_2020_pig_10_eut.	SP10	Spleen ^θ^	15.5
CReSA_2020_pig_11_eut.	S11	Serum	18.6
CReSA_2020_pig_11_eut.	E11	EDTA-blood	16.2
CReSA_2020_pig_11_eut.	B11	Bone-marrow ^θ^	18.8
CReSA_2020_pig_11_eut.	SP11	Spleen ^θ^	17.0
CReSA_2020_pig_12_eut.	S12	Serum	19.1
CReSA_2020_pig_12_eut.	E12	EDTA-blood	15.5
CReSA_2020_pig_12_eut.	B12	Bone-marrow ^θ^	17.4
CReSA_2020_pig_12_eut.	SP12	Spleen ^θ^	15.8
Spleen, 1st passage LPPAM 13-05-2019 1/50	1p19	Cell culture supernatant	27.2

^α^ Samples were obtained from the infection study by Olesen et al. [32]; ^β^ Based on ASFV qPCR analysis as described by Tignon et al. [35]. Ct values above 42 are considered negative (No Ct); ^θ^ 25% *w*/*v* suspensions, see Materials & Methods.

**Table 3:** PCR matrix overview.

Matrix	1× ^α^	10× ^α^	100× ^α^
EDTA-blood	+++	++	+
Bone-marrow	-	++	+
Spleen	-	++	+
Serum	+	-	-

**^α^** Dilution of sample; - no band; + indicates strength of band

## Data Availability

MiSeq data are available at NCBI BioProject with accession no. PRJNA1065849.

## Supplementary Materials

The following supporting information can be downloaded at: https://www.mdpi.com/article/10.3390/pathogens13020154/s1, Figure S1: Deletion screening; Table S1: Overview of SNP analysis of EDTA blood samples Table S2: Overview of Lo-Freq indel analysis of EDTA blood samples; Table S3: Overview of Nanopore indel analysis of EDTA blood samples; Table S4: Overview of Lo-Freq SNP analysis of pig 2 samples. Table S5: Overview of Lo-Freq indel analysis of pig 2 samples. Table S6: Overview of Nanopore indel analysis of pig 2 samples.
