# Supplementary material for: A Deep Sequencing Strategy for Investigation of Virus Variants within African Swine Fever Virus-Infected Pigs
Source: Pathogens. 2024 Feb 8;13(2):154. doi: 10.3390/pathogens13020154 (PMC10893071; doi:10.3390/pathogens13020154)
Supplement: Supplementary file 1 [file pathogens-13-00154-s001.zip › FigureS1.pdf]

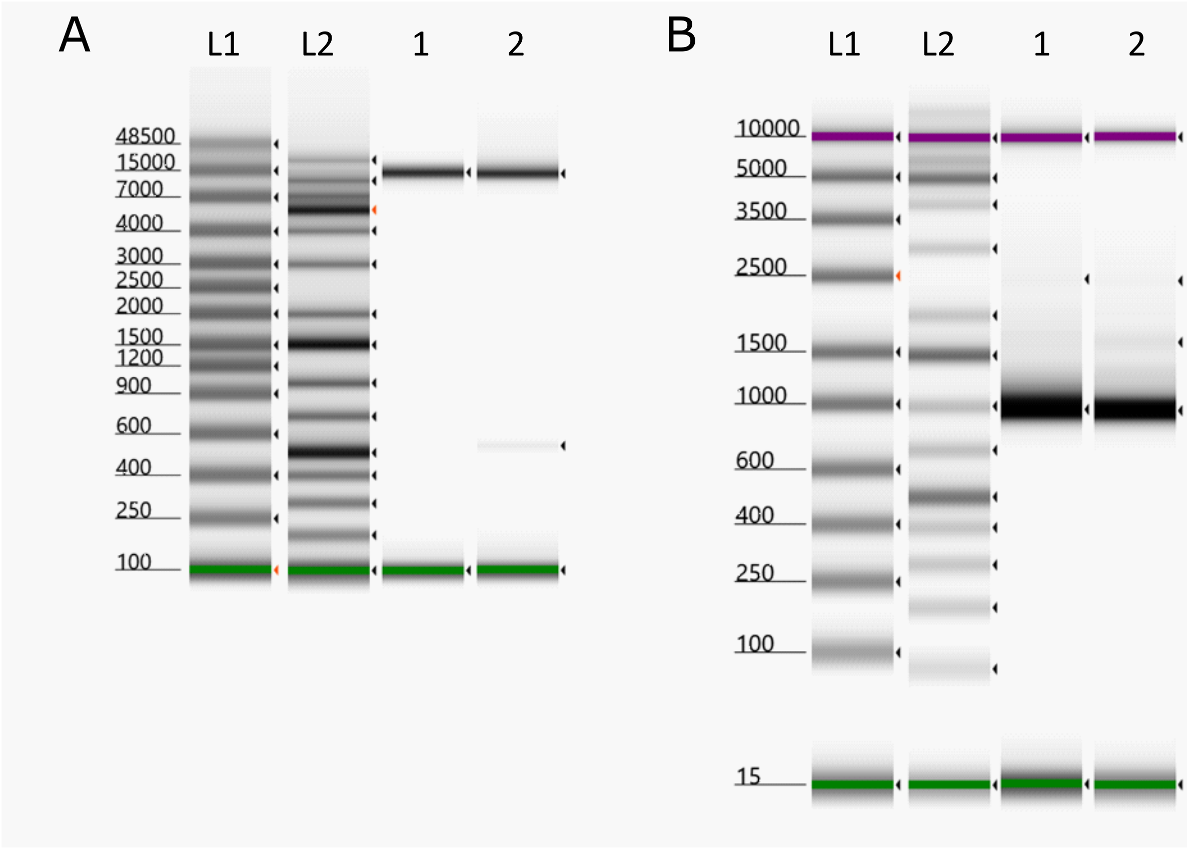

Figure S1. Deletion screening. A) PCRs with primers covering nt 6188-17145. L1: Genomic Ladder. L2: GeneRuler 1kb plus. 1: First passage. 2: Inoculum. B) PCR with primers covering nt 6708-7668. L1: D5000 Ladder. L2: GeneRuler 1kb plus. 1: First passage. 2: Inoculum.
